# Sharing a whole-/total-body [^18^F]FDG-PET/CT dataset with CT-derived segmentations: an ENHANCE.PET initiative

**DOI:** 10.1038/s41597-026-07218-y

**Published:** 2026-04-14

**Authors:** Daria Ferrara, Manuel Pires, Sebastian Gutschmayer, Josef Yu, Yasser G. Abdelhafez, Elisabetta Abenavoli, Ramsey D. Badawi, Abhijit J. Chaudhari, Moon S. Chen, Simon R. Cherry, Armin Frille, Barbara K. Geist, Stefan Gruenert, Marcus Hacker, Swen Hesse, Teresa Kerkhoff, Pia Linder, Johanna Pappisch, Smilla Pusitz, Osama A. Raslan, Ivo Rausch, Siba P. Raychaudhuri, Osama Sabri, Fabian P. Schmidt, Roberto Sciagrà, Benjamin A. Spencer, Guobao Wang, Hubert Wirtz, Thomas Beyer, Lalith Kumar Shiyam Sundar

**Affiliations:** 1 QIMP Team, Medical University of Vienna, Vienna, Austria; 2 Division of Nuclear Medicine, Medical University of Vienna, Department of Biomedical Imaging and Image-guided Therapy, Vienna, Austria; 3 Department of Radiology, University of California Davis, Sacramento, California, USA; 4 Division of Nuclear Medicine, Azienda Ospedaliero Universitaria Careggi, Florence, Italy; 5 Comprehensive Cancer Center, University of California Davis, Sacramento, California, USA; 6 School of Biomedical Engineering and Imaging Sciences, King’s College London, London, UK; 7 Division of Respiratory Medicine, Department of Medicine II, Leipzig University Medical Center, Leipzig, Germany; 8 Department of Nuclear Medicine, University Hospital Leipzig, Leipzig, Germany; 9 Department of Nuclear Medicine and Clinical Molecular Imaging, University Hospital Tübingen, Tübingen, Germany; 10 Department of Medicine and Dermatology, UC Davis School of Medicine, Sacramento, California, USA; 11 Werner Siemens Imaging Center, Department of Preclinical Imaging and Radiopharmacy, University of Tübingen, Tübingen, Germany; 12 DIGIT-X Lab, Department of Radiology, LMU Munich, Munich, Germany

**Keywords:** Computed tomography, Whole body imaging, Positron-emission tomography

## Abstract

We present a large whole-body and total-body curated dataset of dual-modality 2-deoxy-2-[^18^F]fluoro-D-glucose (FDG)-Positron Emission Tomography/Computed Tomography (PET/CT) studies, consisting of 1,683 PET/CT images and the corresponding CT-derived segmentations of 130 target regions. This multi-center dataset includes images from individuals without overt disease and patients with a range of malignant and inflammatory pathologies, including arthritis, lymphoma, and melanoma, as well as cancers of the lung, head-neck, and genito-urinary tract. Target regions were first automatically segmented from CT images using an in-house software and subsequently verified and corrected by physicians-in-training. In total, the segmented regions encompass 130 volumes, including abdominal organs, muscles, bones, cardiac subregions, vessels, adipose tissue, and skeletal muscle around the third lumbar vertebra. PET/CT images and corresponding CT-derived segmentations are provided in anonymized NIfTI format. The dataset can be used for deep learning training, validation, or multi-modality image analysis and thus fills an important gap in available resources to advance the use of PET/CT data in clinical management.

## Background & Summary

In recent years, the field of biomedical engineering and medical physics has witnessed an increase in the complexity of data^1^, driven by rapid advancements in imaging technologies. Traditional data analysis methods have become increasingly inadequate to analyse these large data sets to meet the demand for greater diagnostic precision, and the shift toward personalized treatment strategies^2,3^. To support personalised medicine, more efficient, automated approaches capable of processing and interpreting large-scale datasets are needed. Artificial intelligence (AI) and machine learning (ML) have emerged as powerful tools in this context, offering the ability to identify complex patterns and insights that may not be apparent through conventional methods. However, the effectiveness of AI, particularly of deep learning, is heavily dependent on the availability of large, high-quality and heterogeneous datasets, thus requiring extensive training on vast amounts of data to achieve generalizability and robustness^4^. This limitation is especially acute in nuclear medicine, where publicly available PET/CT datasets with comprehensive anatomical annotations are scarce.

Combined positron emission tomography (PET) and computed tomography (CT) integrates both anatomical and functional imaging capabilities, making it indispensable for diagnosing, staging, and monitoring diseases, such as oncological disorders^5–7^. Despite the clinical importance of PET/CT datasets, open sourcing of imaging data is hindered by strict regulations, and analyses are often conducted in-house^8^ on limited data. At present, very few nuclear medicine datasets with annotated lesions are publicly available: only 1,014 PET/CT lung cancer, lymphoma, melanoma, and healthy control cases from the AutoPET challenge^9^, and another 845 head and neck cancer cases through the HECKTOR challenge^10^. In contrast, Ma *et al*.^11^ identified over one million open-source non-nuclear medicine datasets, most of which originating from radiology and not segmented, including more than 350,000 from CT scans alone. This restricts the development and validation of computational methods for functional imaging, such as image and tumor segmentation, volumetric analysis (e.g., for body composition assessment^12^), and radiomics.

Recent advancements in PET/CT technology, particularly the shift from single-organ imaging^13^ to total-body PET/CT systems^14,15^, allow for simultaneous imaging of multiple organs, fueling multi-organ analyses^16^ and the exploration of systemic metabolic abnormalities^17,18^. However, the development of reliable AI methods for automated analysis of these complex datasets requires access to comprehensive open-source resources, including both images and high-quality segmentations of anatomical structures, which are critical for applications such as diagnosis, treatment planning^19^, volumetric analysis, and patient-specific dosimetry^20,21^.

In the field of CT imaging alone, few open-source datasets include corresponding anatomical segmentations. Rister *et al*.^22^ presented a dataset of 140 abdominal, neck-to-pelvis, and whole-body CT images from patients with liver cancer, segmented into six organ regions. The WORD dataset^23^ comprises 170 abdominal CT images, primarily from prostate, cervical, or rectal cancer cases, along with the segmentations of 16 abdominal organs. These studies, however, are limited in the number of available CT images and segmented regions, and they do not extensively cover different pathologies. More recently, Koitka *et al*. introduced the Sparsely Annotated Region and Organ Segmentation (SAROS) dataset^24^, which consists of 900 abdominal, thoracic, or whole-body CT images from various pathologies. This work focused on 13 semantic body regions and six body parts, including annotations for every fifth image slice. Similar scope and scale were achieved in the AbdomenCT-1k study^25^, which focused on the liver, kidneys, spleen, and pancreas segmentations, and the comprehensive TotalSegmentator dataset^26^, with CT images of the abdomen, pelvis, or thorax segmented into a total of 104 regions of interest.

However, these datasets are limited in scope, often focusing on specific body regions rather than total-body imaging. It is understood that CT images alone are sufficient for many applications, such as volumetric analysis for body composition^12^ or the delineation of organs at risk in radiotherapy treatment planning^27^. In other applications, however, the functional information from PET imaging is essential as it provides complementary insights into disease mechanisms that CT alone cannot offer. For example, in pathological settings, [^18^F]FDG-uptake can help track disease progression by detecting systemic changes in metabolism, such as those seen in patients with infections^28,29^, chronic inflammation^30^, metabolic syndrome^31^ or cancer-associated cachexia^32–34^. In studies involving healthy cohorts, longitudinal [^18^F]FDG PET/CT imaging allows for monitoring metabolic activity in participants and how it changes with aging or other factors^35–37^. Also, a more complete understanding of normal physiological metabolism would help identify deviations that may signal early stages of disease^18^. While the aforementioned AutoPET^9^ and HECKTOR^10^ challenges provide large PET/CT datasets, they focus on segmentations of pathological tissues but ignore healthy anatomical regions. The field currently lacks any open-source PET/CT dataset that provides total-body anatomical segmentations at scale. This gap limits a wide range of PET-centered research efforts that depend on anatomical delineations: without anatomical labels, even basic tasks such as region-specific uptake normalization cannot be evaluated in a reproducible manner across centers. Although CT-derived segmentations do not perfectly correspond to PET signal due to possible motion or misregistration, they provide a reliable anatomical framework to guide PET-based analysis (Figure S1, Supplementary Materials). No existing open-source PET/CT dataset currently provides large-scale, high-resolution anatomical segmentations covering the entire body. This gap becomes even more limiting for total-body PET/CT analyses, where multi-organ metabolic interactions and systemic abnormalities are a topic of growing interest. Resources such as UDPET^38^ provide extensive PET list-mode data and simulated low-dose reconstructions but do not include anatomical segmentations, which inherently restricts their applicability for organ-level or structural analyses, segmentation benchmarking, or multimodal deep learning.

**Fig. 1:**
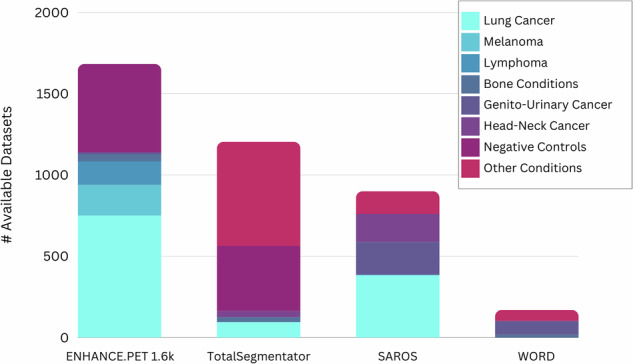
Comparison of clinical indications of cases included in the ENHANCE.PET 1.6k dataset^43^ and other open-source CT images and CT-derived segmentations (TotalSegmentator^26^, SAROS^24^ and WORD^23^). The clinical indications for cases within the TotalSegmentator dataset were derived from the non-imaging parameters provided as a CSV file (https://zenodo.org/records/8367088).

In the present study, we address the limited availability of open-source PET/CT images with segmented tissues as part of our ENHANCE.PET^39^ initiative, which aims to facilitate the sharing of open-source tools and datasets to support research within the PET community. We curated a large [^18^F]FDG PET/CT dataset with anatomical segmentations fully verified by human readers. This dataset includes 1,683 whole-body and total-body PET/CT scans, along with corresponding CT-derived segmentations of 130 non-pathological tissues per scan. The initial segmentations were generated using our in-house tool, MOOSE^40^, for automatic CT segmentation, and were manually verified and corrected using 3D Slicer^41^, a software platform for image analysis. To ensure consistent anonymization, all images underwent a unified defacing procedure. The data includes contributions from the LuCaPET consortium (grant number ERAPerMed_324, “*Clinical decision support for predicting cachexia in cancer patients using hybrid PET/CT imaging*”), the University of California Davis Total-body PET research programme, and from the AutoPET Challenge^9^, whose images and lesion segmentations were already available as open-source on The Cancer Imaging Archive^42^. Focused mainly on the oncological cases of lung cancer, melanoma, and lymphoma (Fig. 1), the ENHANCE.PET 1.6k dataset^43^ also includes head and neck cancer, arthritis, genitourinary cancers, and participants without known disease. The dataset is provided in anonymized NIfTI format to ensure patient privacy, along with demographic details and CT and PET acquisition parameters as non-imaging metadata.

**Fig. 2:**
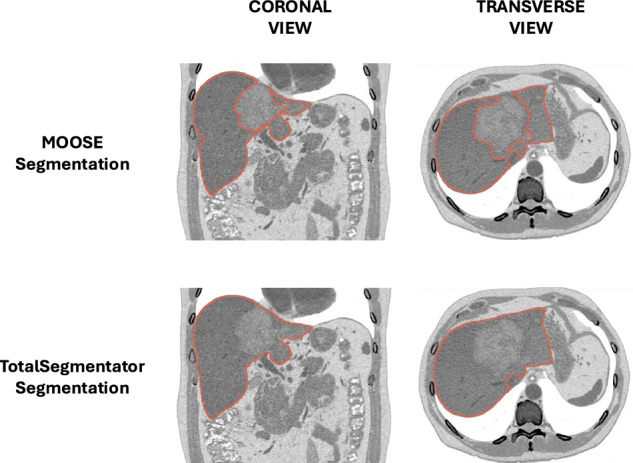
Example of liver segmentation from the “CTLiver” sample data of the 3D Slicer^41^ software. Segmentations were performed using MOOSE^40^, our in-house tool for automatic CT segmentation trained with the ENHANCE.PET 1.6k dataset^43^, as well as TotalSegmentator^26^. Both models accurately segmented the liver volume. However, MOOSE excluded the large liver lesion from the segmentation. In contrast, the output from TotalSegmentator included both healthy and pathological tissue. The ability of MOOSE to differentiate small non-/malignant tissue in low-contrast CT images remains to be studied.

Compared to other publicly available datasets, ENHANCE.PET 1.6k uniquely focuses on the segmentations of organ volumes while avoiding pathological tissues (e.g., tumors, Fig. 2). This emphasis on high-resolution, whole-body anatomical labels makes the dataset particularly valuable for organ-level PET analysis, multi-organ metabolism research, and the development of segmentation methods for the identification of healthy tissues. We believe that the open availability of this dataset will advance the differential understanding of healthy and pathological tissues in computational medicine. This comprehensive resource is now available to facilitate future research and advanced data analysis in whole-body PET/CT imaging. We anticipate that applications such as developing and validating deep learning algorithms for automated data analysis and studies on disease-related systemic abnormalities will greatly benefit from this high-quality data collection.

## Methods

### Data collection

**Fig. 3:**
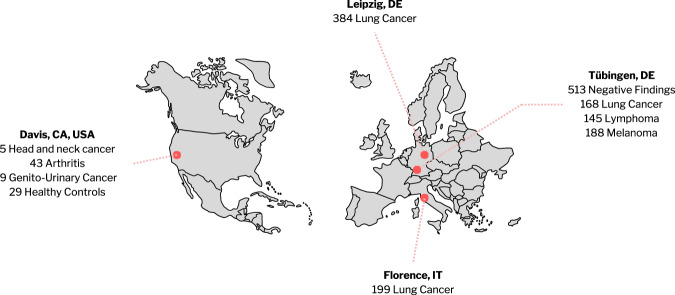
Geographic distributions and clinical indications of the ENHANCE.PET 1.6k dataset^43^. Red dots on the map represent the four clinical facilities of University of California Davis; University of Tübingen, Germany; University Hospital Leipzig, Germany; and Azienda Ospedaliero Universitaria Careggi, Italy.

The ENHANCE.PET 1.6k dataset^43^ was acquired in accordance with the guidelines set forth in the Declaration of Helsinki. All contributing centers provided explicit approval for open, public data release of fully de-identified imaging data. Images were acquired between 1999 and 2022 from various institutions and studies, summarized in Fig. 3: the open-source dataset AutoPET^9^, the University of California Davis in California (IRB: 1721206, 1479228, 1374902, 1341792), the University Hospital Leipzig in Germany (IRB: 259/18-ek and its amendment) and the Azienda Ospedaliero Universitaria Careggi in Italy (IRB: 21306_oss) as part of the LuCaPET consortium. Both the University Hospital Leipzig and the Azienda Ospedaliero Universitaria Careggi obtained institutional review board approval with a waiver of consent for retrospective use and public sharing of anonymized PET/CT data. The cases from the University of California Davis were explicitly authorized for open sharing after application of the standardized defacing and anonymization workflow described below.

Participant demographics across the different clinical conditions are summarized in Table 1.

**Table 1:** Demographics and clinical details of the participants included in the study.

Partner University	Clinical Condition	#	Sex	Age [years]	Weight [kg]	Height [cm]
Azienda Ospedaliero Universitaria Careggi, IT	Lung Cancer	199	73 F / 126 M	71 ± 10	71 ± 16	170 ± 7
University Hospital Leipzig, DE	Lung Cancer	384	113 F / 271 M	65 ± 11	76 ± 16	172 ± 9
Open-source dataset AutoPET^9^	Lung Cancer Lymphoma Melanoma Negative Findings	168 145 188 513	65 F / 103 M 69 F / 76 M 77 F / 111 M 233 F / 280 M	66 ± 9 46 ± 19 65 ± 13 59 ± 15	75 ± 15 77 ± 18 81 ± 21 80 ± 19	171 ± 8 171 ± 10 172 ± 10 172 ± 11
University California Davis, USA	Head and Neck Cancer Arthritis Genitourinary Cancer Healthy Controls	5 43 9 29	1 F / 4 M 12 F / 31 M 1 F / 8 M 16 F / 13 M	N/A 54 ± 15 67 ± 9 47 ± 13	88 ± 21 93 ± 20 79 ± 13 80 ± 17	171 ± 10 173 ± 9 176 ± 7 170 ± 10

### Imaging protocols

Five different PET/CT systems were used for image acquisition at the participating medical centres: United Imaging Healthcare uEXPLORER (Number of acquired scans = 86), Siemens Biograph mCT (N = 1014), Siemens Biograph 16 (N = 384), Philips Gemini TF (N = 180), and GE Healthcare Discovery MI (N = 19). At all four sites, diagnostic CT scans were acquired with X-ray tube voltages between 100 kVp and 140 kVp, and CT data were reconstructed with a slice thickness between 1 mm and 5 mm. Details on the CT reconstruction parameters are provided in Table 2.

**Table 2:** Summary of imaging systems and CT reconstruction parameters of the ENHANCE.PET 1.6k dataset^43^.

Partner University	Azienda Ospedaliero Universitaria Careggi, IT N = 199	University Hospital Leipzig, DE N = 384	Open-source dataset AutoPET^9^ N = 1014	University California Davis, USA N = 86
PET/CT System Manufacturer	GE Medical System (19) Philips (180)	Siemens	Siemens	United Imaging Healthcare
System Model	Discovery MI (19) Gemini TF TOF 16 (180)	Biograph 16	Biograph mCT	uEXPLORER
kVp	120 (196) 140 (3)	120	100 (2) 120 (938) 140 (74)	140
Filter Type	N/A	NONE	FLAT	Default
Convolutional Kernel	N/A STANDARD (19)	B10f (325) B31f (56) B40f (3)	I30f (31) I31f (471) B30f (24) B31f (488)	BodySharp (43) BodySoft (43)
Axial Pixel Size (mm)	0.98–1.37	0.98	0.69–0.98	0.49–1.37
Slice Thickness (mm)	3.75 (19) 5 (180)	2 (36) 3 (347)	1 (52) 2 (141) 3 (821)	1 (43) 2.34 (42) 3 (1)
Focal Spot Size (mm)	N/A	0.7 (330) 1.2 (54)	1.2	1

Participants were asked to fast for 6 hours before the examinations and were scanned in the supine position, with arms up in the medical facilities in Italy and Germany and arms down at the University of California, Davis. Each subject underwent a static PET acquisition following an intravenous injection of [^18^F]FDG (306 ± 66 MBq). Uptake times varied across the four sites, with an average of (68 ± 29) minutes post-injection. PET images were reconstructed with attenuation and scatter corrections applied using the corresponding CT data.

Details on the CT and PET acquisition parameters are reported for each participant as non-imaging parameters in the available spreadsheet files. The download link is provided in the Data Records section.

### Segmentations and data processing

**Fig. 4:**
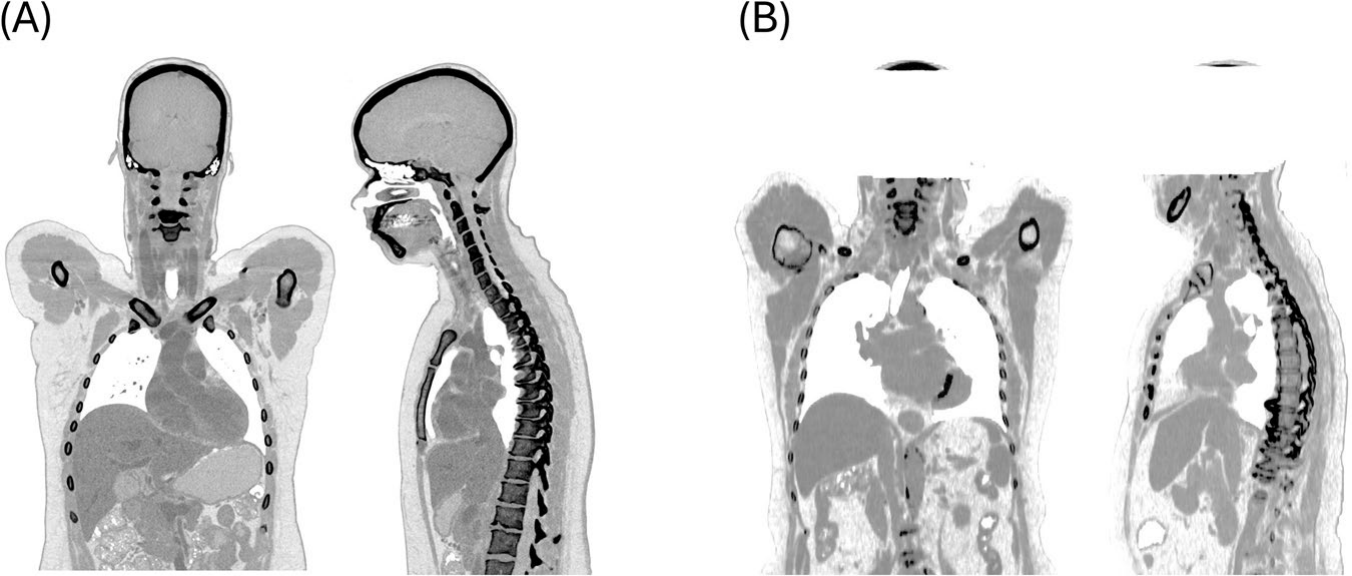
Coronal and sagittal views of (**A**) an original CT image from the AutoPET Challenge^9^ and (**B**) an anonymized CT image, defaced, from the Azienda Ospedaliero Universitaria Careggi, Italy. Defacing of the PET/CT images was performed as an additional measure for complete anonymization of patients prior to data open-sourcing.

PET/CT images were retrieved in anonymized DICOM format from the participants and centralized at the Medical University of Vienna. The metadata were used to extract relevant information about the CT and PET acquisition protocols as well as essential demographic details of the participants. For subsequent analysis and segmentation, all data were converted to NIfTI format using the *dcm2niix* DICOM to NIfTI converter^44^. To ensure uniform anonymization across centers and that participants could not be visually identified from their CT images^45–47^, both the PET and CT images from all sites were edited: in the PET images, voxels between the upper part of the skull segmentation and the bottom of the brain, within a cylinder of 16 voxels in the z-direction, were set to zero (Fig. 4B). Similarly, the corresponding CT region was set to -1000 Hounsfield Units to simulate air. This same standardized defacing pipeline was applied to all datasets prior to public release.

To maximize efficiency and accelerate the workflow, the processing of the entire ENHANCE.PET 1.6k dataset^43^ was done serially: automatic segmentation of the CT images, manual refinement of the derived labels, and retraining of the original segmentation models, according to the following scheme. Our in-house developed software, MOOSE^40^, was first used for the automatic segmentation of 384 lung cancer images from the University Hospital Leipzig. The resulting segmentations were manually refined by 10 medical students using the 3D Slicer image analysis software^41^. For each dataset, a student was randomly assigned to verify and correct the segmentations, addressing possible systematic errors such as inaccuracies at anatomical borders of target regions, mislabelling between left and right regions, or misclassification of regions with similar intensities on CT. A second student was then tasked with reviewing the first student’s work and correcting any remaining mistakes. Once both students agreed on the final version of the dataset, it was reviewed by a radiology resident and a nuclear medicine resident. The PET images overlapped with the corresponding CT images were used to exclude pathological tracer-avid regions from organ masks to ensure that segmentations represent healthy tissue only.

**Fig. 5:**
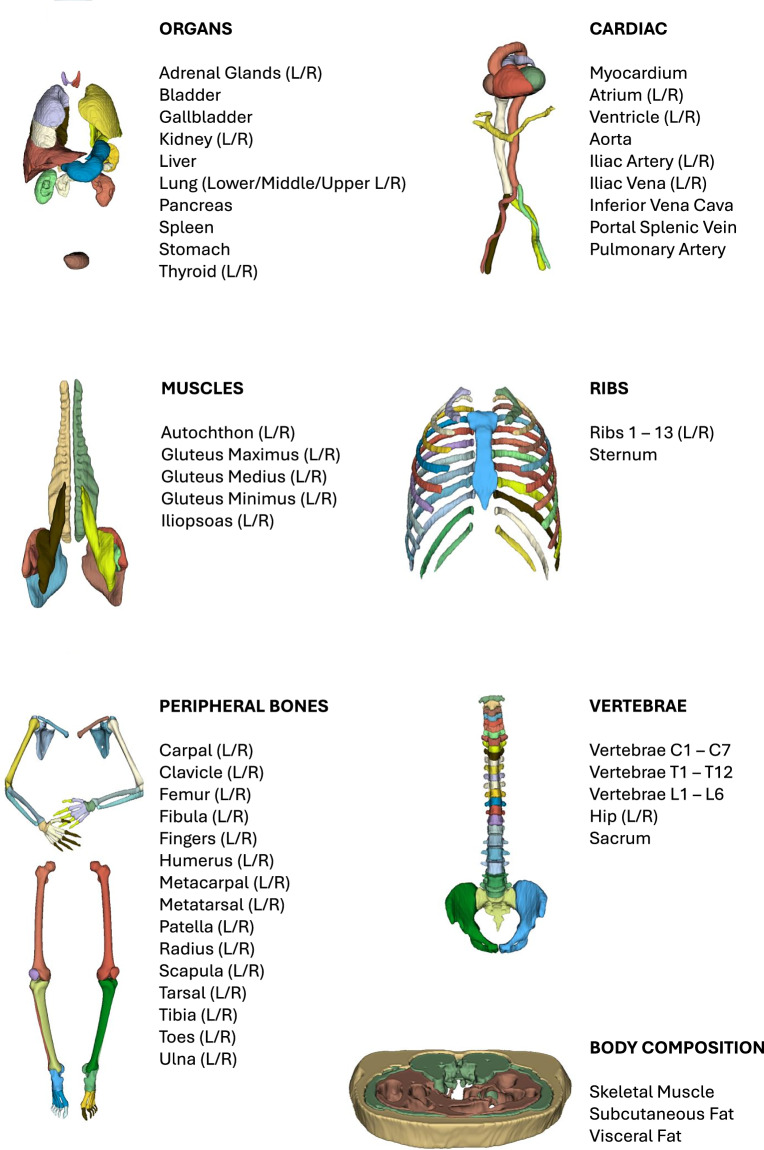
Complete list of segmented target regions per dataset. L = left; R = right.

The segmentations were organized into seven anatomical groups, as shown in Fig. 5: organs, cardiac, muscles, ribs, peripheral bones, vertebrae, and body composition around the L3 vertebra volume. Separate nnU-Net^48^ models were trained for each anatomical group. Details on the retraining process are provided in the Technical Validation section.

The newly trained models were subsequently used for the segmentation of the second dataset, originating from Azienda Ospedaliero Universitaria Careggi, and again underwent manual refinement and quality control described above. The same workflow of automatic segmentation, manual refinement, and model retraining was then applied to the remaining images from the University of California Davis and the AutoPET^9^ open-source dataset. In the case of the AutoPET data, since the corresponding lesion segmentations were available online, they were taken as ground truth and directly subtracted from the organ segmentations without the need for manual correction.

**Fig. 6:**
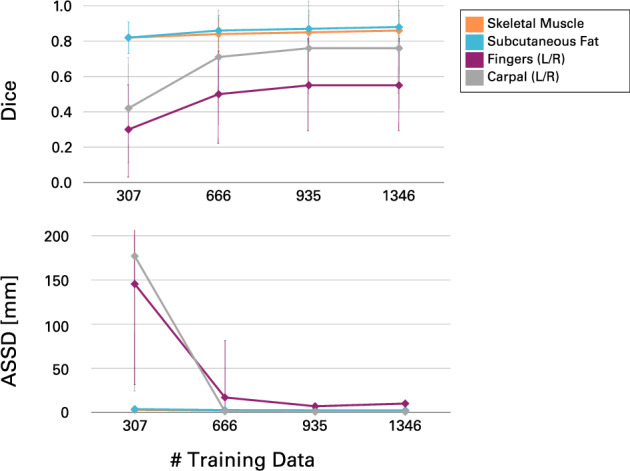
Performance of the CT-segmentation models on selected target regions as a function of the training dataset size. Performance was evaluated on 20% of the ENHANCE.PET 1.6k dataset^43^ (N = 337), as described in the Technical Validation section. As the training dataset size increased, DICE scores for segmentation improved, primarily due to the correction of systematic segmentation errors, such as left/right misclassification in the hands and the inclusion of missing regions in the “skeletal muscle” segmentation. Similarly, the average symmetric surface distance (ASSD) decreased as the dataset size grew.

At each stage of retraining, the size of the training data increased, improving model performance. This iterative process allowed for efficient verification and faster corrections by the medical students without compromising precision, especially in regions where systematic errors had been identified and were hindering the manual correction process. Segmentation of the fingers and hand bones showed the most significant improvement as the training dataset grew (Fig. 6). In the first round of training, several instances of left-right misclassification were identified, especially when the hands were crossed over the abdomen or above the head. However, this issue progressively improved with each retraining step. Another improvement achieved through more extensive training data was the automatic inclusion of the quadratus lumborum muscle in the “skeletal muscle” label for body composition, which had previously been missing and required manual correction (Fig. 6).

## Data Record

The PET/CT images and corresponding segmentations are hosted on the Science Data Bank (ScienceDB, https://www.scidb.cn/en). The dataset can be downloaded either via command line following MOOSE^40^ installation, as described in the Code Availability section, or directly through the associated 10.57760/sciencedb.34150.

**Fig. 7:**
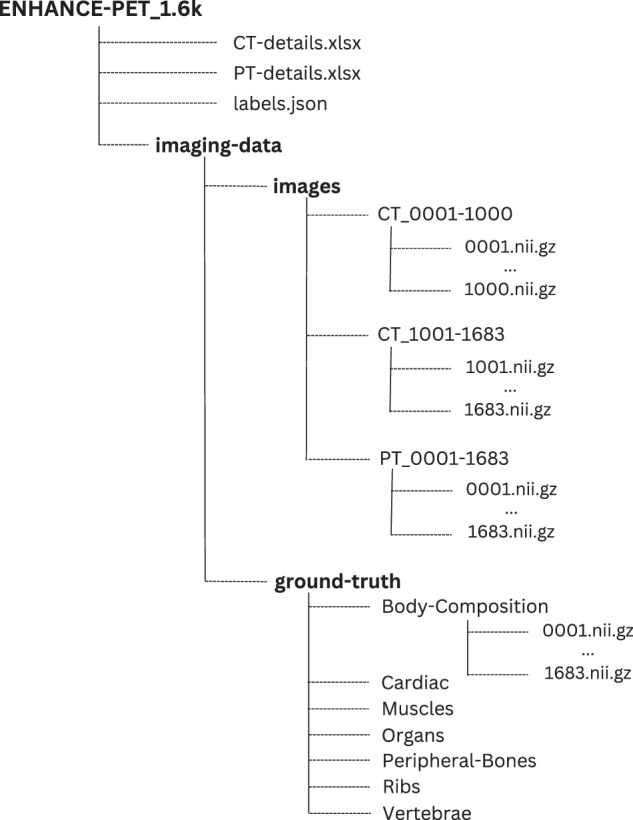
Folder structure of the ENHANCE.PET 1.6k dataset^43^. Each image and segmentation are provided in anonymized NIfTI format and named with ascending unique IDs from 0001.nii.gz to 1683.nii.gz. For each participant, the CT image, PET image, and segmentations of cardiac subregions and vessels, muscles, organs, peripheral bones, ribs, vertebrae, and body composition (including skeletal muscle and adipose tissue) around the L3 vertebra region are provided.

The imaging data are stored in separate folders containing the CT images, PET images, and ground truth segmentations, respectively. Within the segmentations folder, there are seven subfolders corresponding to the different segmentation classes listed above: “Body-Composition,” “Cardiac,” “Muscles,” “Organs,” “Peripheral Bones,” “Ribs,” and “Vertebrae.” Each folder contains NIfTI data files, named sequentially from 0001.nii.gz to 1683.nii.gz. Due to file size limitations, the CT images are divided into two folders: the first contains files 0001.nii.gz to 1000.nii.gz, and the second contains files 1001.nii.gz to 1683.nii.gz. A JSON file containing the complete list of segmentations and their corresponding intensities within the multi-class files is also available for download. The directory structure of the ENHANCE.PET 1.6k dataset^43^ is shown in Fig. 7.

On some Linux and macOS systems, the downloaded compressed folders may occasionally be assigned restrictive file permissions, which can prevent users from opening or modifying the files after extraction. In this case, updating the folder and files permissions (e.g., via a right-click “Get Info” menu or an equivalent command-line operation) resolves the issue and allows normal access.

In addition to the imaging data, non-imaging information is provided in two spreadsheet files. The *CT-details.xlsx* file contains details on the CT acquisition parameters (e.g., PET/CT system manufacturer and model, kVp, filter type, convolutional kernel, axial pixel size, slice thickness, and focal spot size) for each participant. The *PT-details.xlsx* file provides the corresponding demographic information (e.g., clinical indication, sex, age, weight, height) as well as PET acquisition parameters (e.g., injected activity, acquisition date and time, radioactivity injection details, image units, slope, intercept, system model and manufacturer).

## Technical Validation

We used the ENHANCE.PET 1.6k dataset^43^ to develop a deep learning-based method for the automatic segmentation of CT scans. We performed a 80/20 train/test split, preserving the proportion of images per center and clinical condition: 1,346 images for training and 337 for testing. Seven separate nnU-Net models were trained for each anatomical group: (i) organs, (ii) cardiac tissues, (iii) muscles, (iv) ribs, (v) peripheral bones, (vi) vertebrae/sacrum, and (vii) body composition at L3. A detailed list of regions segmented by each model is shown in Fig. 5.

Prior to training, all images and labels were resampled with SimpleITK (https://simpleitk.org/about.html) from the original resolution to a voxel spacing of 1.5 × 1.5 × 1.5 mm using B-spline interpolation, whereas segmentation masks were resampled using nearest-neighbor interpolation to preserve discrete label boundaries. At inference, input CTs are resampled to 1.5 mm isotropic spacing; predicted masks are resampled back to native resolution for storage and evaluation. Training was run for 2,000 epochs using the open-source nnU-Net framework, without mirroring-based augmentation except for the body-composition task. Training configuration files and instructions are provided in the MOOSE GitHub repository (https://github.com/ENHANCE-PET/MOOSE/blob/main/planning_training_ENHANCE.PET.md) to enable full reproducibility.

**Fig. 8:**
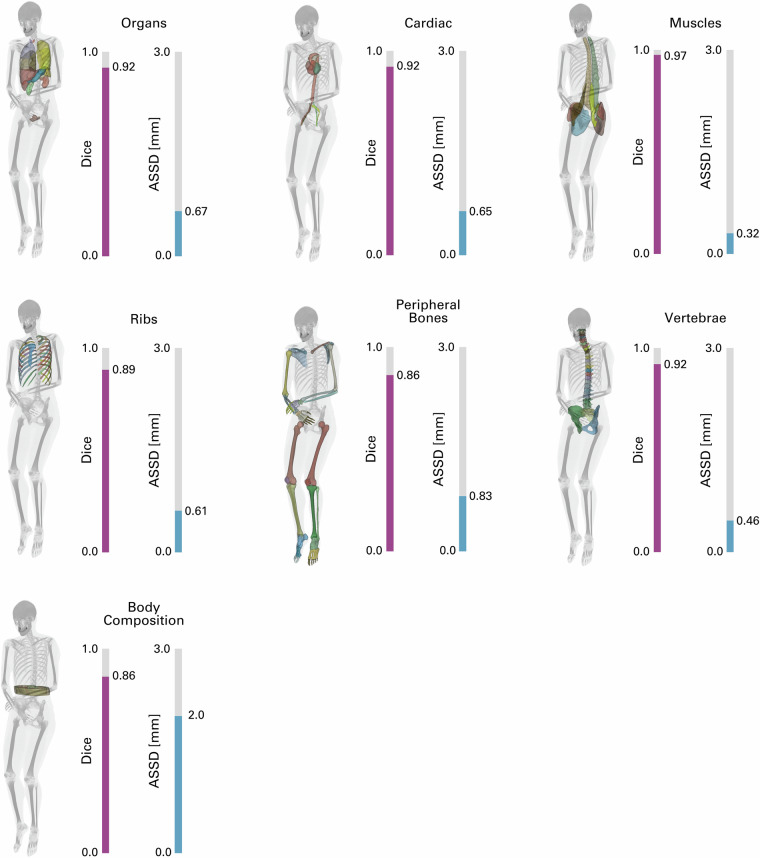
Mean Dice scores and Average Symmetric Surface Distance (ASSD) per available segmentation model between reference labels of the test dataset (N = 337, 20% of the total ENHANCE.PET 1.6k dataset^43^) and the labels resulting from MOOSE^40^ prediction.

To assess the model performance, we tested all segmentation models on the remaining 20% of the ENHANCE.PET 1.6k. Segmentation accuracy was evaluated using the Dice Similarity Coefficient (DSC) to quantify the overlap between predicted segmentations and the reference labels and with the Average Symmetric Surface Distance (ASSD)^49^ to estimate the average distance between surface voxels of the reference labels and the automated segmentation. The averaged results for the generated models are shown in Fig. 8, and the metrics for each label are reported in Table S1, Supplementary Materials.

All models achieved high accuracy, with mean DSC values exceeding 0.85 across most regions and mean ASSD values below 3 mm in all regions. The “Muscles” model achieved the highest overlap and the lowest prediction error, with an average DSC of 0.97 ± 0.02 and an ASSD of 0.3 ± 0.2 mm (Fig. 8). Cardiac, organ, and vertebrae models also achieved high average DSC values, exceeding 0.90. The peripheral bones model had the lowest performance, with an average DSC of 0.86 ± 0.27 and the highest variation in ASSD, at 0.8 ± 7.5 mm. The lower performance in these regions is likely due to their small size and thin anatomical structures: the digits of the hand had the most significant negative impact on model performance, with some cases of left/right misclassification identified (especially when patients underwent imaging with their hands crossed over the abdomen), resulting in an average DSC of 0.55 ± 0.39 and an ASSD of 10 ± 37 mm (Table S1, Supplementary Materials). Similarly, the segmentation of the metacarpals yielded a DSC of 0.71 ± 0.33 and an ASSD of 5 ± 23 mm. Other regions with lower overlap included the portal and splenic veins (DSC: 0.82 ± 0.18) and the adrenal glands (DSC: 0.82 ± 0.12), most likely due to their low contrast resolution in CT imaging of the test dataset, which makes delineation more challenging. The ribs and body composition models also showed higher variation in ASSD, at 0.6 ± 2.1 mm and 2.0 ± 1.7 mm, respectively.

The ENHANCE.PET 1.6k dataset^43^ proved to be suitable for training models for automated CT image segmentation. This dataset has the potential to contribute significantly to further advancements in deep learning-based approaches, including attempts to improve segmentation models performance or the addition of new volumes of interest not covered in the present study. The dual availability of both CT and PET images, together with the inclusion of segmentations for multiple anatomical regions, makes the ENHANCE.PET 1.6k dataset particularly valuable for research focused on diseases that affect multiple organs or systems, such as metabolic disorders or systemic inflammatory diseases^50,51^, or for studies on normal glucose metabolism in healthy tissues.

A limitation of the dataset is that all anatomical segmentations were derived from the CT images. As a result, the correspondence between CT-based labels and PET images may be compromised in cases of significant patient motion, which was not systematically assessed across the entire cohort. To provide an estimate of the potential impact of this effect, we performed an additional analysis on two representative cases exhibiting mild and severe respiratory motion (Figure S1, Supplementary Materials). In these cases, liver segmentations derived from CT were compared with PET-based liver segmentations, and the resulting standardized uptake values (SUVs) were quantified. For the case with mild motion, the difference in extracted SUV was approximately 3%, whereas for the case with severe motion the difference increased to 16% (mean SUV for PET-based segmentation = 2.2 ± 0.7; mean SUV for CT-based segmentation = 2.6 ± 0.5). Although the latter represents a larger deviation, the extracted mean SUV remains within the range of variability reported in the literature for [^18^F]FDG-PET quantitative measurements^52^. Therefore, we believe that CT-based segmentations can be safely used for PET analyses in most cases, while remaining mindful of potential PET–CT misalignment issues, particularly in the presence of pronounced patient motion.

To ensure consistent anonymization across centers, a uniform defacing procedure was applied to all PET and CT volumes. This process removes facial structures, including brain and skull regions, and therefore limits the direct applicability of the dataset to neuroimaging or cranial analyses. Users interested in brain-specific PET or CT studies should take this aspect into account when selecting the dataset for their applications.

Within these defined boundaries, the ENHANCE.PET 1.6k dataset^43^ provides a large-scale, multi-center resource for the development and evaluation of automated segmentation methods and PET/CT analysis workflows, particularly for whole-body and multi-organ studies. We hope that this open-source dataset will accelerate developments in medical imaging, ultimately contributing to the advancement of personalized medicine and more effective clinical decision-making.

## Usage Notes

All imaging data are presented in NIfTI format, ensuring participants’ privacy while allowing for easy use in further analysis. This format can be opened with most visualization software, including 3D Slicer (https://www.slicer.org/) and ITK-SNAP (http://www.itksnap.org/pmwiki/pmwiki.php). DICOM to NIfTI conversion was performed using *dcm2nii*^44^, and all image processing was conducted using Python.

## Supplementary information

Supplementary Materials

## Data Availability

The PET/CT images and corresponding ground-truth segmentations from the ENHANCE.PET 1.6k dataset are publicly available in the Science Data Bank (ScienceDB) repository at the following 10.57760/sciencedb.34150. The dataset includes CT images, PET images, and multi-class segmentation masks stored in NIfTI format, organised into folders corresponding to anatomical segmentation classes. A JSON file listing the segmentation labels and their corresponding intensity values within the multi-class files is also provided. In addition to the imaging data, two spreadsheet files containing non-imaging metadata are available: *CT-details.xlsx*, which includes CT acquisition parameters for each participant, and *PT-details.xlsx*, which contains participant demographic information and PET acquisition parameters. The dataset can be downloaded directly from the repository via the DOI link above or via command line following installation of MOOSE^40^, as described in the Code Availability section.

## Code availability

The segmentation software MOOSE^40^ and the presented open-source dataset are part of the ENHANCE.PET (https://enhance.pet/) initiative for facilitating data sharing and collaboration within the PET community.

MOOSE code is open-source and available online with extensive documentation, and can be accessed on GitHub at https://github.com/ENHANCE-PET/MOOSE. Following MOOSE installation within a Python environment, the dataset can be downloaded via the command line: < moosez -dtd -dd path/to/download/ >. All code used for dataset preparation and segmentation follows the nnU-Net framework^48^, a publicly available and well-established open-source pipeline for biomedical image segmentation (https://github.com/MIC-DKFZ/nnUNet). No additional custom code beyond this framework was used in the dataset generation process. As described in the Technical Validation section, training configuration files and instructions are also provided in the MOOSE GitHub repository (https://github.com/ENHANCE-PET/MOOSE/blob/main/planning_training_ENHANCE.PET.md).
