# Supplementary material for: Sharing a whole-/total-body [18F]FDG-PET/CT dataset with CT-derived segmentations: an ENHANCE.PET initiative
Source: Sci Data. 2026 Apr 14;13:869. doi: 10.1038/s41597-026-07218-y (PMC13249809; doi:10.1038/s41597-026-07218-y)
Supplement: Supplementary file 1 — Supplementary Materials [file 41597_2026_7218_MOESM1_ESM.docx]

### **Supplementary Materials**

**Figure S1.** Representative coronal and transverse views of the CT (upper line) and PET (bottom line) images from a case exhibiting severe respiratory motion. In all panels, the CT-derived liver segmentation is shown in red, while the PET-derived liver segmentation is shown in green. Clear spatial discrepancies between the two segmentations are visible due to PET–CT misregistration.

### **Table S1**. Mean Dices and Average Symmetric Surface Distance (ASSD) per segmented volume between the reference labels of the test dataset (N=337, 20% of the total ENHANCE.PET 1.6k dataset) and the labels resulting from the AI prediction. Left and right regions were merged for this analysis.

| **Model** | **Regions** | **Mean Dice ± St Dev** | **Mean ASSD ± St Dev [mm]** |
| --- | --- | --- | --- |
| Organs | Adrenal Glands  Bladder  Gallbladder  Kidney Liver  Lung Lower Lobe  Lung Middle Lobe  Lung Upper Lobe  Pancreas  Spleen  Stomach  Thyroid | 0.82 ± 0.12  0.90 ± 0.18  0.88 ± 0.15  0.96 ± 0.05 0.98 ± 0.03  0.95 ± 0.12  0.94 ± 0.09  0.97 ± 0.05  0.90 ± 0.08  0.96 ± 0.08  0.95 ± 0.06  0.87 ± 0.19 | 0.5 ± 0.5  1.0 ± 2.0  0.8 ± 1.7  0.5 ± 0.7 0.6 ± 0.5  0.6 ± 1.4  0.8 ± 1.8  0.9 ± 0.6  0.7 ± 0.7  0.7 ± 0.8  0.7 ± 1.0  0.6 ± 0.8 |
| Cardiac | Myocardium  Atrium  Ventricle  Aorta  Iliac Artery  Iliac Vena  Inferior Vena Cava  Portal Splenic Vein  Pulmonary Artery | 0.92 ± 0.05  0.96 ± 0.03  0.96 ± 0.03  0.95 ± 0.02  0.90 ± 0.09  0.92 ± 0.07  0.92 ± 0.06  0.82 ± 0.18  0.94 ± 0.05 | 0.5 ± 0.4  0.5 ± 0.4  0.5 ± 0.4  0.5 ± 0.2  0.6 ± 1.9  0.8 ± 1.8  0.6 ± 0.5  0.9 ± 1.8  0.8 ± 0.6 |
| Muscles | Autochthon  Gluteus Maximus  Gluteus Medius  Gluteus Minimus  Iliopsoas | 0.98 ± 0.01  0.98 ± 0.01  0.98 ± 0.01  0.96 ± 0.03  0.97 ± 0.02 | 0.3 ± 0.2  0.3 ± 0.2  0.3 ± 0.2  0.3 ± 0.2  0.3 ± 0.2 |
| Ribs | Rib 1  Rib 2  Rib 3  Rib 4  Rib 5  Rib 6  Rib 7  Rib 8  Rib 9  Rib 10  Rib 11  Rib 12  Sternum | 0.86 ± 0.12  0.88 ± 0.12  0.88 ± 0.11  0.90 ± 0.10  0.90 ± 0.09  0.91 ± 0.09  0.91 ± 0.09  0.91 ± 0.09  0.90 ± 0.10  0.90 ± 0.11  0.89 ± 0.14  0.86 ± 0.18  0.94 ± 0.03 | 0.5 ± 0.6  0.4 ± 0.8  0.4 ± 0.6  0.4 ± 0.9  0.5 ± 2.0  0.5 ± 1.8  0.6 ± 2.4  0.6 ± 2.3  0.7 ± 1.9  0.8 ± 2.7  0.9 ± 3.2  0.9 ± 3.7  0.5 ± 1.1 |
| Vertebrae | Vertebra C1  Vertebra C2  Vertebra C3  Vertebra C4  Vertebra C5  Vertebra C6  Vertebra C7  Vertebra T1  Vertebra T2  Vertebra T3  Vertebra T4  Vertebra T5  Vertebra T6  Vertebra T7  Vertebra T8  Vertebra T9  Vertebra T10  Vertebra T11  Vertebra T12  Vertebra L1  Vertebra L2  Vertebra L3  Vertebra L4  Vertebra L5  Hip  Sacrum | 0.89 ± 0.10  0.92 ± 0.09  0.91 ± 0.09  0.90 ± 0.09  0.90 ± 0.11  0.90 ± 0.09  0.91 ± 0.09  0.93 ± 0.09  0.93 ± 0.10  0.92 ± 0.10  0.93 ± 0.09  0.93 ± 0.07  0.93 ± 0.08  0.93 ± 0.09  0.94 ± 0.08  0.94 ± 0.08  0.94 ± 0.10  0.94 ± 0.11  0.94 ± 0.12  0.93 ± 0.13  0.93 ± 0.14  0.93 ± 0.15  0.93 ± 0.16  0.90 ± 0.20  0.97 ± 0.08  0.96 ± 0.08 | 0.5 ± 0.7  0.4 ± 0.4  0.4 ± 0.4  0.4 ± 0.5  0.4 ± 0.4  0.4 ± 0.4  0.4 ± 0.6  0.4 ± 0.7  0.4 ± 0.9  0.4 ± 0.9  0.4 ± 0.9  0.4 ± 0.5  0.4 ± 0.6  0.4 ± 1.0  0.4 ± 0.9  0.5 ± 1.0  0.5 ± 1.2  0.5 ± 1.4  0.6 ± 1.7  0.7 ± 2.2  0.7 ± 2.4  0.7 ± 2.6  0.8 ± 2.6  0.6 ± 2.0  0.3 ± 0.2  0.3 ± 0.2 |
| Peripheral Bones | Carpal  Clavicle  Femur  Fibula  Fingers  Humerus  Metacarpal  Metatarsal  Patella  Radius  Scapula  Tarsal  Tibia  Toes  Ulna | 0.76 ± 0.33  0.91 ± 0.16  0.96 ± 0.15  0.93 ± 0.19  0.55 ± 0.39  0.94 ± 0.15  0.71 ± 0.33  0.92 ± 0.17  0.92 ± 0.19  0.66 ± 0.42  0.91 ± 0.16  0.95 ± 0.17  0.95 ± 0.16  0.90 ± 0.15  0.68 ± 0.40 | 0.8 ± 0.6  0.5 ± 2.1  0.3 ± 0.6  0.3 ± 0.5  10.0 ± 37.5  0.4 ± 1.0  5.2 ± 22.7  0.8 ± 4.1  0.5 ± 1.0  0.5 ± 0.8  0.3 ± 0.4  1.4 ± 4.5  0.3 ± 0.3  0.6 ± 1.3  0.5 ± 0.6 |
| Body Composition | Skeletal Muscle  Subcutaneous Fat  Visceral Fat | 0.85 ± 0.08  0.87 ± 0.08  0.87 ± 0.09 | 2.0 ± 1.5  2.4 ± 1.8  1.5 ± 1.6 |
